# Prevalence, attitudes, and practices of dietary supplements among middle-aged and older adults in Asir region, Saudi Arabia: A cross-sectional study

**DOI:** 10.1371/journal.pone.0292900

**Published:** 2023-10-12

**Authors:** Amani Alhazmi, Beena Briget Kuriakose, Sakeena Mushfiq, Khursheed Muzammil, Manal Mohammed Hawash

**Affiliations:** 1 Department of Public Health, College of Applied Medical Sciences, King Khalid University, Abha, Saudi Arabia; 2 Department of Basic Medical Science, College of Applied Medical Sciences, King Khalid University, Abha, Saudi Arabia; 3 Department of Gerontological Nursing, College of Nursing, Alexandria University, Alexandria, Egypt; King Abdulaziz University Faculty of Medicine, SAUDI ARABIA

## Abstract

The objectives of this study were to 1) identify the prevalence of dietary supplements (DS) among middle-aged and older adults; 2) determine attitudes toward DS and patterns of DS usage among middle-aged and older adults; and 3) assess the association of sociodemographic, clinical, and lifestyle factors with DS attitudes and DS usage. A cross-sectional online survey was undertaken among middle-aged and older adults. Data were collected by an online self-administered pretested questionnaire used as a study tool and distributed to respondents via social media applications. The DS usage prevalence among 501 respondents was 50.7%, and 53.5% of participants reported a positive attitude toward DS. The positive attitudes and higher usage of dietary supplements were statistically significant in higher proportions among older adults compared to middle-aged individuals. Significantly lower proportion of middle aged (54.9%) reported taking DS daily compared to 59.9% of older adults. A significant difference in the type of DS among the two groups was found for Omega-3 (p<0.001), minerals (p = 0.004), proteins (p = 0.002), fibers (p = 0.002), phytonutrients (p = 0.007), and probiotics (p = 0.015), with a higher proportion of middle-aged respondents reporting their use compared to older adults. Dietary supplement usage is a prevalent phenomenon among older adults and the middle-aged population. However, some undesirable practices regarding their use still exist in the community. Thus, there is a need of focussed health education to enhance attitudes and improve practices regarding the use of DS.

## Introduction

Diet and nutrition have a direct role in the maintenance of good health and prevention of diseases. Although a well-balanced diet is expected to provide essential nutrients, the role of dietary supplements (DS) in complementing the diet cannot be overlooked [1]. DS are defined as products, not drugs that contain an ingredient intended to supplement the diet. They include vitamins, minerals, and other less familiar substances, as herbals, amino acids, and enzymes. Although these products often contain 100% or more of the daily need of one or more nutrients, they are not meant to replace the variety of foods that are important to a healthful diet. In addition, DS are different from drugs and are not intended to treat, diagnose, prevent, or cure diseases [2]. In high-income countries, DS occupy a well-established, scientifically proven position in correcting malnutrition and are also perceived to be helpful in preventing style life diseases, extending life expectancy, and improving life quality [3]. The use of DS has been on steady increase during the last decade. DS use in adults has been consistently reported to increase with age, income, and education [4]. Previous studies have shown that DS users are more likely to adopt a number of positive health-related habits. These include better dietary patterns, exercising regularly, maintaining a healthy body weight, and avoidance of tobacco products [4]. Scientific research in this area have indicated a number of reasons behind DS use as to maintain good health, ensure adequate nutrition, improve physical appearance and to achieve weight loss [2].

As people age, activity levels and energy requirements tend to decrease. Concomitant decreases in food consumption may cause protein and micronutrient intake to fall below desired levels [5]. During the middle adulthood (40 to 60 years), people begin to experience the first outward signs of ageing such as changes in the digestive system, joint aches, loss of muscle tone and elasticity wear and tear injuries etc. [6]. In the older adults (above 60 years), poor nutrition can result from inappropriate food intake; poverty; social isolation; multiple and/or chronic medication use; decreases in functional status; changes in physiology that affect digestion, absorption, and/or metabolism of nutrients; variety of diseases or oral health problems or emotional impairment [5]. In the above context, there is a scope and probability of use of DS among middle aged and older adults. While DS may contribute to achieving nutritional adequacy, they have not shown to benefit in the primary prevention of chronic disease, including cardiovascular disease, cancer, or mortality [7]. In addition, lack of sufficient regulation of DS raises concerns for contamination, improper dosing, drug-supplement interactions, lack of efficacy, and unestablished safety profiles [7]. Besides these, a considerable number of people consuming DS do not seek any medical advice before taking them [8]. However, the use of DS is on the increase because they are readily available, cost-effective and consumer- compliant [9]. Globally, over the past 40 years, there has been an observed increase in the use of DS from 25–70% [10]. A lot of research has been conducted on the knowledge and use of DS among university students [11–13] and its use among athletes [14–16]. However, very little research has been conducted on the various aspects of DS among middle aged and older adults. Hence, the current research is carried out with the following objectives. 1. To assess the knowledge attitude, and practice of DS among middle aged and older adults. 2. To assess the association of socio-demographic factors with knowledge, attitude, and practice of DS.

## Materials and methods

### Study design

A cross-sectional survey was conducted among middle-aged (45-< 60 years) and older adults (60 years and over) residing in Asir Province, southwest of the Kingdom of Saudi Arabia. The age group and residence in Asir region were the main eligibility criteria. Given the high internet usage among people in the KSA, data were collected online via a self-administered pretested questionnaire using Google forms. A link to the survey was distributed to respondents via social media applications (Twitter and WhatsApp groups). Exclusion questions were posted before the start of questionnaire to ensure that the survey opens only for eligible respondents. 87 participants were excluded at the preliminary stage. The sample size was calculated using the standard formula SS = 4pq/l2, taking p = 55.75% [17] and a relative error of 8% [18]. The overall calculated sample size was 496. In total, 501 participants were included in the analysis. Study subjects were chosen by convenience sampling. The questionnaire was piloted among thirty participants from the target population to assess the questionnaire’s face validity and ensure that the questions were understandable. The pilot study responses were excluded from the primary analysis. Data collection continued for a month during June 2021. Participation of the study subjects in this study was voluntary, and the questionnaire was filled out anonymously.

### Questionnaire

A self-administered 28-item pretested questionnaire including an interface and three sections was used as a study tool for data collection. The questionnaire interface explained the study’s aims and informed participants that their participation was voluntary. Informed consent was obtained before proceeding with the questionnaire. To ensure data confidentiality, no personal identifiers were collected. The required fields option was used to avoid missing data. The first section focused on general sociodemographic, anthropometric, and lifestyle information. The second part explored attitudes toward the usage of dietary supplements. A 5-point Likert scale ranging from (1) strongly disagree to (5) strongly agree was used to categorize the responses. Survey items in this area asked participants about the importance of DS for all ages and whether they perceived dietary supplements to be generally harmless or have a possible preventive role in chronic disease, as well as respondents’ beliefs related to the statement "supplements are not necessary when following a healthy diet" and the potentially harmful side effects and interaction potential with other supplements or medications. The third section addressed DS practices, including frequency of nutritional supplement use, sources of information, types of nutrition supplements, and reasons for taking dietary supplements. Content validity was achieved by administering the questionnaire to nine nutrition and public health experts, as well as those in related fields. The Arabic version of the questionnaire was measured for internal reliability using Cronbach’s alpha correlation coefficient. The results exhibited good internal consistency, stability, and reliability (α = 0.965).

### Statistical methods

The collected data was checked twice for their correctness and completeness. The data were coded and analysed for practical statistical significance using Microsoft Excel and IBM SPSS Statistics for Windows, Version 21.0. Armonk, NY: IBM Corp. Descriptive statistics such as the frequency, mean, standard deviation, median, and coefficient of variation were used to describe the quantitative variables. Descriptive analysis, including means and standard deviations, was used to analyse data on continuous dependent and independent variables. The categorical data were described by percentages. A Chi-Square test was employed to examine the association between the categorical independent variables and nominal levels, with significance levels of 0.05 and 0.01.

### Ethical considerations

The study was approved by the King Khalid University ethical committee (Approval No. ECM#2022–1003). Informed consent was obtained from all participants involved in the study using the interface of the online questionnaire *via* a statement that informed the respondents that their response assumed that they provided informed consent. To ensure data confidentiality, no personal identifiers were collected.

## Results

Most respondents among middle-aged and older adults were males (60.5% and 64.6%, respectively), married (90.4% and 89.1%, respectively), educated at least up to university (68.6% and 59.9%, respectively), and had sufficient income (55.1% and 60.5%, respectively). Further, there were more employed (52.8% and 46.3%) than unemployed (19.8% and 23.8%) and retired (27.4% and 29.9%) individuals in both age categories, as shown in Table 1.

**Table 1:** Socio-demographic characteristics of the studied middle aged and older adults (N = 501).

Socio-demographic characteristics	Middle Aged		Older Adults	
	(45 - < 60 years)		(≥ 60 years)	
	(n = 354)		(n = 147)	
	No	%	No	%
**Gender**				
Male	214	60.5	95	64.6
Female	140	39.5	52	35.4
**Marital status**				
Married	320	90.4	131	89.1
Single	34	9.6	16	10.9
**Educational level**				
Pre-University	111	31.4	59	40.1
University or Higher	243	68.6	88	59.9
**Monthly Income**				
Sufficient	195	55.1	89	60.5
Insufficient	159	44.9	58	39.5
**Employment status**				
Employed	187	52.8	68	46.3
Unemployed	70	19.8	35	23.8
Retired	97	27.4	44	29.9

Table 2 indicates that among middle-aged study participants, 42.9% reported having a chronic disease compared to 49.7% among the older adults. Diabetes Mellitus was the most commonly reported pre-existing chronic disease among both groups. Similarly, most of the respondents in both age groups were non-smokers (77.7% among middle-aged adults and 90.7% among older adults). In total, 50.2% of older adults reported daily physical activity compared to 30.2% of middle-aged adults. Notably, 22.6% of middle-aged respondents and 19.0% of older adults reported having no physical activity. In both age groups, the majority reported a regular sleeping pattern (78.8% middle aged and 89.1% older adults). Among middle-aged participants, 23.7% were overweight, and 15.5% were obese, while among older adults, 15% were overweight, and 12.9% were obese.

**Table 2:** Clinical and lifestyle characteristics of the studied middle aged and older adults (N = 501).

Clinical and lifestyle characteristics	Middle Aged		Older Adults	
	(n = 354)		(n = 147)	
	No	%	No	%
**Do You have any Chronic Disease?**				
Yes	152	42.9	73	49.7
No	202	57.1	74	50.3
**If yes, what are they?**	**(n = 152)**		**(n = 73)**	
Hypertension	58	38.2	25	34.2
Type 2 diabetes	95	62.5	53	72.6
Heart disease	12	7.9	6	8.2
Musculoskeletal disease	31	20.4	15	20.5
Respiratory diseases	28	18.4	5	6.8
Kidney diseases	8	5.3	3	4.1
Liver diseases	5	3.3	2	2.7
Neurological disorders	7	4.6	2	2.7
**Smoking status**				
Non-Smoker	275	77.7	133	90.5
Smoker	79	22.3	14	9.5
**Physical Activity**				
Daily (at least 30 minutes)	107	30.2	74	50.3
Occasional (less than twice a week)	167	47.2	45	30.6
No physical activity	80	22.6	28	19.0
**Adequate sleep (at least 6 hours/day)**				
Yes	279	78.8	131	89.1
Occasional	40	11.3	13	8.8
No	35	9.9	3	2.0
**Nutritional status (Body Mass Index)**				
Normal (18.5–24.9)	215	60.7	106	72.1
Overweight (25.0–29.9)	84	23.7	22	15.0
Obese (30.0 and above)	55	15.5	19	12.9

**Fig 1:**
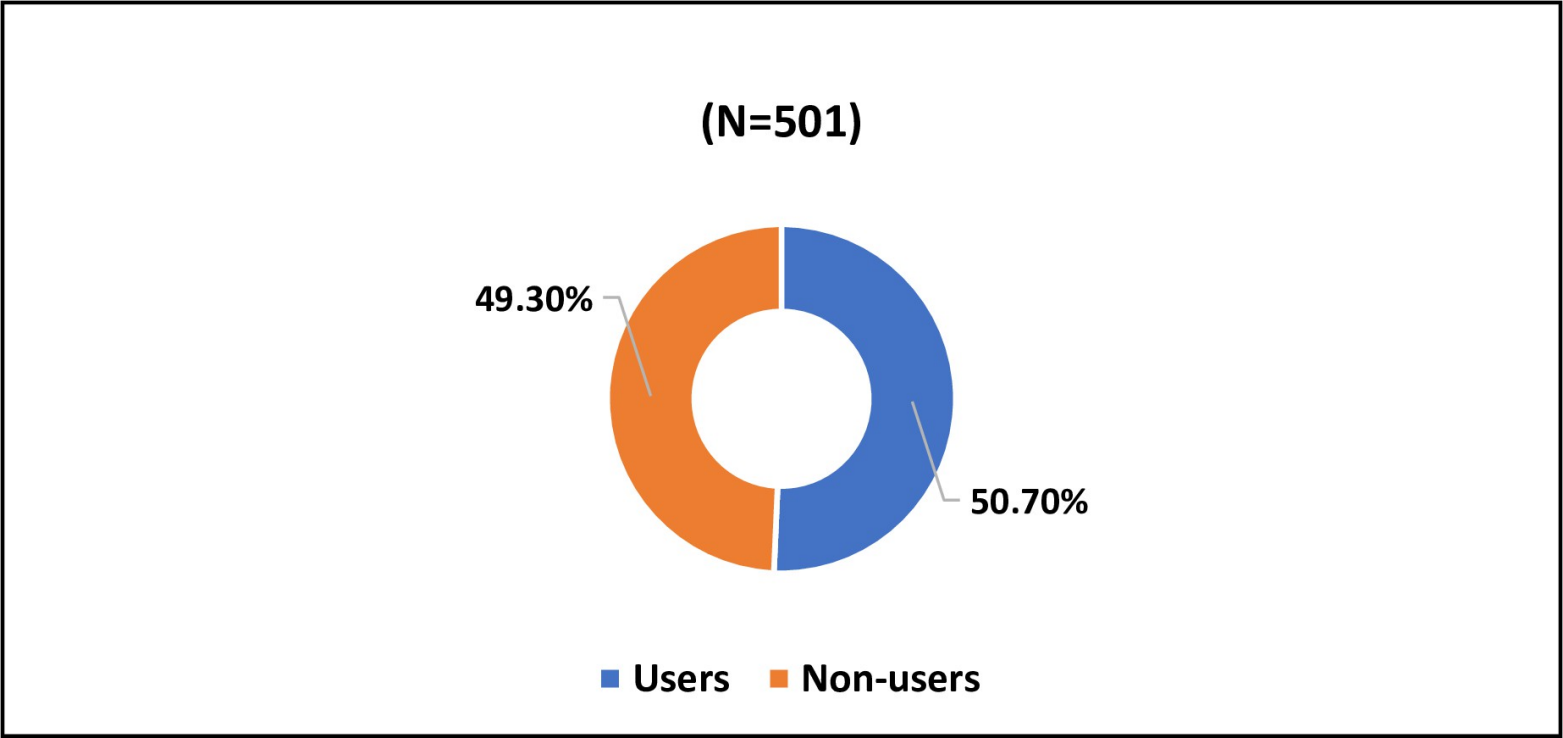
Dietary supplements usage among the subjects.

**Fig 2:**
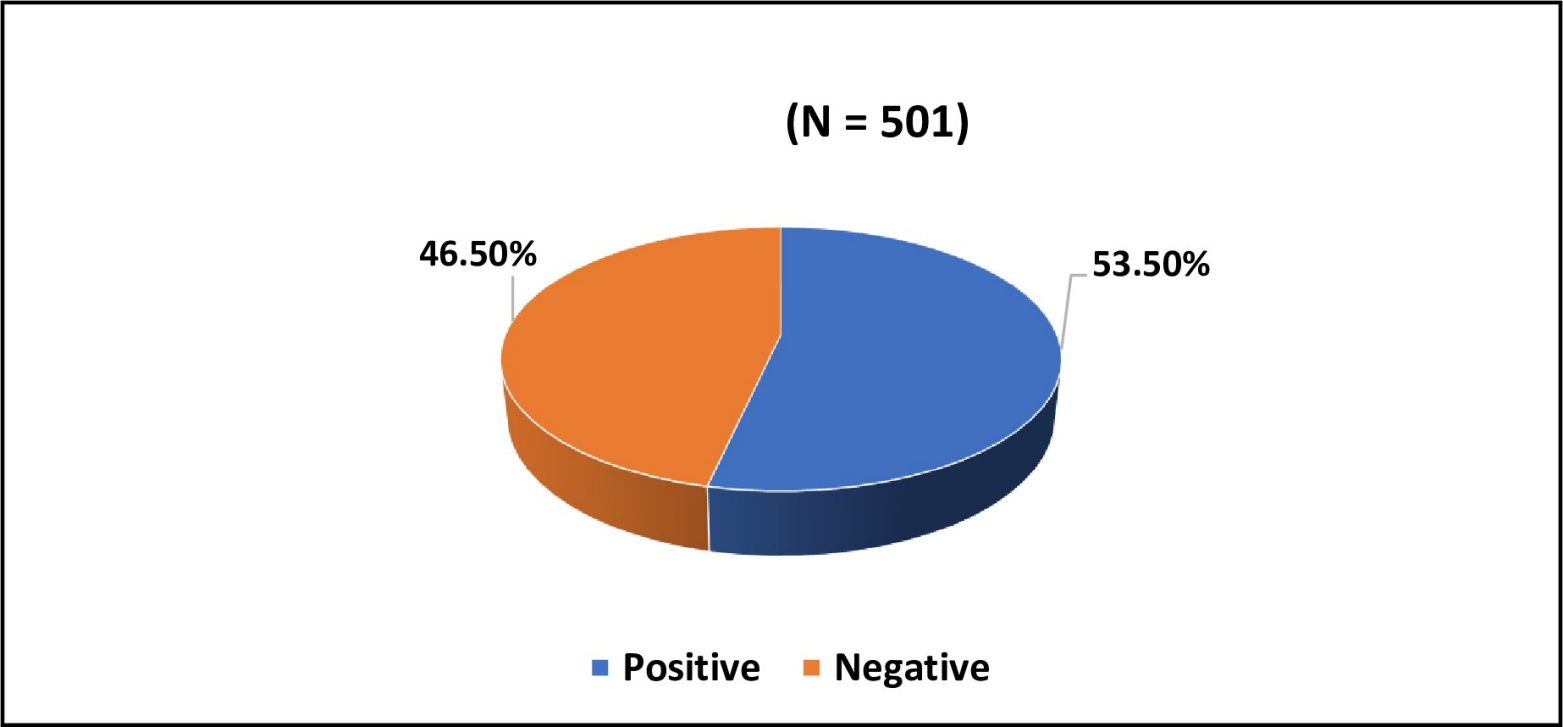
Attitude toward dietary supplements among the subjects.

A total of 50.7% of respondents reported using some form of dietary supplements, whereas 49.3% reported that they were non-dietary supplement users (Fig 1). Positive attitude towards DS was reported by 53.5%, whereas 46.5% reported negative attitude towards DS (Fig 2).

This study found a significantly higher usage of DS among older adults (62.6%) compared to middle-aged adults (45.8%) with a statistically significant difference [χ2 = 11.76; p<0.001]. Correspondingly, positive attitudes towards DS were found in a significantly higher proportion of older adults (68%) compared to middle-aged adults (47.5%) with a statistically significant difference [χ2 = 17.66; p<0.001], as shown in Table 3.

**Table 3:** Dietary supplement usage and attitudes among middle- aged and older adults (N = 501).

Dietary supplement usage and attitudes	Middle Adults (n = 354)		Older Adults (n = 147)		Chi-Square	
	**NO**	**%**	**No**	**%**	**χ** ^**2**^	**P**
**DS Usage**						
DS Users	162	45.8	92	62.6	**11.760**	**<0.001** **
DS Non-users	192	54.2	55	37.4		
**DS Attitudes**						
Negative	186	52.5	47	32.0	**17.665**	
Positive	168	47.5	100	68.0		**<0.001** **

** p<0.01

Table 4 compares the pattern of DS usage among middle aged and older adults. Regarding the frequency of DS usage, the study found that a significantly lower proportion of middle aged (54.9%) reported taking DS daily compared to 59.9% of older adults. This difference was found to be statistically significant [χ2 = 6.938; p<0.05]. Additionally, the majority of participants reported using DS “to maintain good health” with statistically significant differences between middle-aged and older adults (46.3% and 72.8%, respectively). Moreover, a significantly lower proportion of older adults (4.3%) reported taking DS “as per a doctor’s advice” compared to middle-aged adults (15.4%) [χ2 = 35.784; p<0.001]. A significant difference in the types of DS consumed among the two groups was found for omega -3 (p<0.001), minerals (p = 0.004), proteins (p = 0.002), fibres (p = 0.002), phytonutrients (p = 0.007), and probiotics (p = 0.015), with a higher proportion of middle-aged respondents reporting their use compared to older adults. Multivitamins were the most common type of DS consumed in both age groups, with no statistically significant difference. Side effects from DS were reported by none of the older adults compared to 6.2% of middle-aged people [χ2 = 20.38; p<0.001]. Notably, a significantly higher proportion of older adults (80.4%) reported not following instructions regarding DS usage compared to middle-aged adults (46.3%) [χ2 = 28.2; p<0.001].

**Table 4:** The pattern of dietary supplements usage among middle-aged and older adults (N = 254).

Pattern of dietary supplements usage	Middle Aged		Older Adults			Chi-Square / Fisher True Test	
	(n = 162)		(n = 92)				
	No	%	No	%	χ^2^		P
**Frequency of DS usage**							
Daily	89	54.9	56	59.9			
Occasional	73	45.1	36	39.1	**6.938**		**0.031** *
**Reason of DS usage** ^**#**^							
As per the doctor’s advice	25	15.4	4	4.3			
To reduce weight	8	4.9	2	2.2			
To ensure adequate nutrition	21	13.0	19	20.7			
To maintain good health	75	46.3	67	72.8	**35.784**		**<0.001** **
To meet increased energy need	10	6.2	0	0.0			
To prevent disease	12	7.4	0	0.0			
No specific reason	11	6.8	0	0.0			
**kind of DS usage#**							
Vitamins	113	69.8	60	65.2	0.556		0.456
Minerals	70	43.2	23	25.0	**8.384**		**0.004** ^*^
Protein	20	12.3	1	1.1	**9.807**		**0.002** ^*^
Conjugated linoleic acid	3	1.9	0	0.0	1.724		0.189
Omega 3	61	37.7	11	12.0	**19.078**		**<0.001** **
Multivitamin/mineral	32	19.8	23	25.0	0.952		0.329
Phytonutrients	12	7.4	0	0.0	**07.153**		**0.007** *
Probiotic	10	6.2	0	0.0	**5.912**		**0.015** ^*^
Fiber	19	11.7	1	1.1	**9.160**		**0.002** ^*^
**DS side effects**							
Yes	10	6.2	0	0.0			
No	152	93.8	92	100	**20.382**		**<0.001** **
**Follow DS instructions**							
Yes	87	53.7	18	19.6			
No	75	46.3	74	80.4	**28.200**		**<0.001** **

* p< 0.05

** p<0.01

# More than one response

As shown in Table 5, gender was found to be statistically significantly associated with DS usage, with the proportion of male DS users being greater than that of females among both middle-aged and older adults and a higher proportion of males among older adults compared to middle-aged individuals [χ2 = 20.368; p<0.01]. The proportion of married DS users was statistically significantly higher than the proportion of single DS users among both middle-aged and older adults. The majority of married DS users were middle-aged [χ2 = 3.895; p<0.05]. DS users in both age categories were also found to have a significantly lower proportion of smokers compared to non-smokers, with the highest proportion of non-smokers being older adults with a statistically significant difference [χ2 = 18.086; p<0.01]. Physical activity was found to be statistically significantly associated with DS usage, with a higher percentage of DS users among both middle-aged and older adults reporting daily physical activity compared to those who reported no physical activity [χ2 = 28.922; p<0.01]. Further, a significantly higher proportion of DS users in both age groups reported adequate sleeping patterns compared to those who reported inadequate sleeping patterns, most of whom were older adults with a statistically significant difference [χ2 = 12.335; p<0.01]. Additionally, the prevalence of obesity was found to be significantly lower among DS users in both age groups compared to those who reported overweight and normal BMIs, the highest percentage of whom were older adults with a statistically significant difference [χ2 = 11.899; p<0.01]. Moreover, a higher proportion of DS users reported positive attitudes toward DS usage in both age groups compared to those who reported negative attitudes, the highest percentage of whom were older adults with a statistically significant difference [χ2 = 15.312; p<0.01].

**Table 5:** Association between dietary supplements usage, sociodemographic, clinical and lifestyle characteristics and attitudes among middle-aged and older adults (N = 254).

Sociodemographic, clinical and lifestyle characteristics and attitudes level	Dietary Supplements Users					Chi–Square / Fischer’s exact test		
	Middle Aged		Older Adults					
	(n = 162)		(n = 92)					
	No	%		No	%		χ^2^	P
**Gender**								
Male	97	59.9		80	87.0			
Female	65	40.1		12	13.0		**20.368**	**<0.001** **
**Marital status**								
Married	150	92.6		78	84.8			
Single	12	7.4		14	15.2		**3.895**	**0.048** *
**Educational level**								
Pre-University	49	30.2		30	32.6			
University or Higher	113	69.8		62	67.4		0.152	0.695
**Monthly Income**								
Sufficient	82	50.6		50	54.3			
Insufficient	80	49.4		42	45.7		0.327	0.567
**Employment status**								
Employed	79	48.8		43	46.7			
Unemployed	31	19.1		23	25.0			
Retired	52	32.1		26	28.3		1.280	0.527
**Chronic Disease**s	58	35.8		44	47.8		3.529	0.060
**Smoking status**								
Non-Smokers	130	80.2		91	98.9			
Smokers	32	19.8		1	1.1		**18.086**	**<0.001** **
**Physical Activity**								
Daily (at least 30 minutes)	80	49.4		61	66.3			
Occasional (less than twice a week)	52	32.1		19	20.7			
No physical activity	30	18.5		12	13.0		**28.922**	**<0.001** **
**Adequate sleep (at least 6 hours/ day)**								
Yes	126	77.8		86	93.5			
Occasional	22	13.6		6	6.5			
No	14	8.6		0	0.0		**12.335**	**0.002** **
**Body Mass Index (BMI)**								
Normal (18.5–24.9)	101	62.3		76	82.6			
Overweight (25.0–29.9)	39	24.1		12	13.0			
Obese (30.0 and above)	22	13.6		4	4.3		**11.899**	**0.002** **
**Attitude toward DS**								
Negative	51	31.5		9	9.8			
Positive	111	68.5		83	90.2		**15.312**	**<0.001** **

* p< 0.05

** p<0.01

As shown in Table 6, positive attitudes toward DS were found to be statistically significantly associated with gender, with the proportion of male DS users being greater than that of female users among both middle-aged and older adults. The highest proportion for males was observed among the older adult subjects [χ2 = 8.062; p<0.01]. Being married was also statistically significantly associated with having a positive attitude toward DS among middle-aged and older adults, among whom the highest proportion of married subjects were middle-aged rather than older adults [χ2 = 3.895; p<0.05]. Positive attitudes toward DS in both age categories were also found to correspond to a significantly lower proportion of smokers compared to non-smokers, among whom the highest proportion of non-smokers was older adults with a statistically significant difference [χ2 = 13.746; p<0.01]. Physical activity was found to be statistically significantly associated with positive attitudes toward DS in both age groups, the highest percentage of whom reported daily physical activity rather than no physical activity; the highest percentage of was older adults with a statistically significant difference [χ2 = 21.824; p<0.01]. Further, a significantly higher proportion of subjects with positive attitudes toward DS in both age groups reported adequate sleeping patterns compared to those who reported inadequate sleeping patterns; the highest percentage was older adults with a statistically significant difference [χ2 = 12.879; p<0.01]. Additionally, the prevalence of obesity was found to be significantly lower in both age groups with positive attitudes toward DS than those who reported overweight and normal BMIs, most of whom were older adults compared to middle-aged adults with a statistically significant difference [χ2 = 9.047; p<0.01].

**Table 6:** Association between dietary supplements attitudes and sociodemographic, clinical and lifestyle characteristics among middle-aged and older adults (n = 254).

Sociodemographic, clinical and lifestyle characteristics	Positive attitude toward DS				Chi–Square / Fischer’s exact test	
	Middle Aged		Older Adults			
	(n = 162)		(n = 92)			
	**No**	**%**	**No**	**%**	**χ** ^**2**^	**P**
**Gender**						
Male	99	58.9	76	76.0		
Female	69	41.1	24	24.0	**8.062**	**0.004** **
**Marital status**						
Married	158	94.0	85	85.0		
Single	10	6.0	15	15.0	**6.066**	**0.013** *
**Educational level**						
Pre-University	58	34.5	34	34.0		
University or Higher	110	65.5	66	66.0	0.007	0.930
**Monthly Income**						
Sufficient	84	50.0	69	69.0		
Insufficient	84	50.0	31	31.0	9.237	0.002
**Employment status**						
Employed	87	51.8	45	45.0		
Unemployed	35	20.8	25	25.0		
Retired	46	27.4	30	30.0	1.223	0.542
**Presence of chronic Disease**	68	40.5	49	49.0	1.851	0.173
**Smoking status**						
Non-Smoker	131	78.0	95	95.0		
Smoker	37	22.0	5	5.0	**13.746**	**<0.001** **
**Physical Activity**						
Daily (at least 30 minutes)	55	32.7	62	62.0		
Occasional (less than twice a week)	72	42.9	24	24.0		
No physical activity	41	24.4	14	14.0	**21.824**	**<0.001** **
**Adequate sleep** (at least 6 hours/day)						
Yes	135	80.4	95	95.0		
Occasional	18	10.7	5	5.0		
No	15	8.9	0	0.0	**12.879**	**0.002** **
**Body Mass Index (BMI)**						
Normal (18.5–24.9)	100	59.5	77	77.0		
Overweight (25.0–29.9)	41	24.4	16	16.0		
Obese (30.0 and above)	27	16.1	7	7.0	**9.047**	**0.011** *

* p< 0.05

** p<0.01

## Discussion

Adequate nutrition and a balanced diet are essential for maintaining health. Due to increasing awareness among people about health and preventing diseases, DS are increasing in popularity [19, 20]. Various studies show that there has been a dramatic increase dietary supplement usage among the general population in developed countries [19, 21, 22]. Against this background, the present study was conducted to gain insight into the prevalence of dietary supplement usage among middle-aged and older adults and its associated factors in Asir region, Saudi Arabia.

The most notable finding of this study is that more than one-half of the participants reported themselves to be DS users and to have positive attitudes toward DS. Positive attitudes and higher usage of DS were statistically significantly more common among older adults compared to middle-aged adults. These findings may be due to chronic illnesses being more prevalent among older adults, which might result in an inadequate consumption of nutrients. Age-related changes such as altered taste perception and teeth loss could lead to reduced food intake, subsequently making older adults vulnerable to vitamin B12 and D deficiencies [5]. Moreover, the higher prevalence of DS users among the elderly may be related to barriers such as limited access to nutritionists and a healthy diet. This is consistent with a study conducted in KSA by El Afifi, which revealed that the prevalence of DS usage was 55.75% [17]. Additionally, the majority of users (94.44%) reported positive attitudes towards nutritional supplements. Similarly, findings of other studies reported that more than half of the participants were DS users and had positive DS attitudes [2, 23]. Several studies also supported these findings and revealed that a higher proportion of DS users were older rather than middle-aged adults [7, 23–26].

Nutrition plays a vital role in maintaining health and preventing diseases. While a well-balanced diet aims to provide essential nutrients, the role of DS in complementing one’s diet cannot be underestimated [20, 27]. DS represent a vital source of essential nutrients [20, 28]. Many public health experts have considered DS at the international level as an essential strategy for the prevention of micronutrient deficiencies and the reduction of congenital disabilities [26, 27]. DS have expanded explosively during recent decades [27]. Multivitamins are the most common supplements used in developed countries. The usage of multivitamins has increased, particularly among older individuals, over the past decades [29]. However, some people may benefit most from taking vitamin and mineral supplements because of their distinct nutritional needs. Women of childbearing age usually need extra iron and calcium. Pregnant or lactating women, older adults, vegetarians, people with eating disorders or medical conditions, and people who often eat processed or fast food are all groups of concern [26]. The present study confirmed this fact and concluded that multivitamins are the most consumed DS category in both age groups, with no statistically significant difference. However, significant differences were found in the types of DS used among the two groups, with a higher proportion of middle-aged respondents reporting the use of Omega-3, minerals, proteins, fibres, phytonutrients, and probiotics compared to older adults. These results are in line with several previous studies [1, 2, 7, 20, 29, 30].

Most participants in the current study reported that they consume DS to "maintain good health" or to "ensure adequate nutrition" for a statistically significantly higher proportion of older rather than middle-aged adults. These results are congruent with the findings of similar studies [1, 2, 20, 24, 25, 31]. Although the use of DS has been linked to adverse reactions, most users worldwide claim that DS cause no harm [24, 32]. The present research confirmed this hypothesis and found that a statistically significantly higher proportion of older adults, compared to middle-aged individuals, reported taking DS daily, with no side effects, despite not following DS usage instructions. This result is consistent with the results found in other studies [24, 25, 32, 33].

Surprisingly, the current study’s findings indicated that the male gender was statistically significantly associated with higher DS usage than the female gender among both middle-aged and older adults. These findings may be related to the higher needs of males for mineral and protein supplements to boost energy and enhance immunity and sexual virility. This finding agrees with a study done by Shahwan MS and Al Abdin, which revealed that males (56.1%) were more likely than females (43.9%) to be DS users [30]. Similarly, El Khoury D and Antoine-Jonville reported that females composed a smaller proportion of supplement users than males [34]. Contrary to these results, multiple studies have found that women are more likely than men to use supplements within each age group [1, 2, 4, 7, 25, 35]. In a study by Alhashem et al., it was observed that women and men varied in their attitudes toward DS usage, in which women had more DS positive attitudes than men, which is inconsistent with our results, where men were statistically significantly associated with higher positive DS attitudes than women among both middle-aged and older adults [23].

The current study also revealed that being married was statistically significantly associated with higher DS usage and positive attitudes towards DS among both middle-aged and older adults. These findings may be related to the influence of the socio-cultural environment in which married partners encourage and support each other to use protein and herbal supplements to enhance their marital relationship. This is congruent with the results of Alhashem et al., who found that married partners were more likely than single participants to use DS. However, the same study revealed that single participants reported more positive attitudes toward DS than married subjects, with a statistically significant difference [23].

Overall, a high proportion of the population uses DS in their routines [30]. Numerous studies have examined lifestyle associations with dietary supplement use. Evidence from numerous surveys showed that dietary supplement users are more likely to adopt numerous healthy behaviours, including daily exercise, good sleeping patterns, an average body weight, and the avoidance of smoking [2, 4, 7, 23, 25, 29, 31, 35]. Dietary supplement users were also more likely to pay attention to their lifestyle patterns. This result was consistent with our findings, where daily physical activity, good sleeping patterns, the avoidance of smoking, and an average body weight were statistically significantly associated with higher DS usage among middle-aged and older adults.

In interpreting the results of this study, some limitations should be considered. This was a cross-sectional study with inherent limitations. Further, this study relied on a self-reported questionnaire, which may have introduced recall bias. Despite the sample size being large enough to support the generalization being made, the use of the convenience sampling method could limit the generalizability of the study. There is also possibility of volunteer bias; people with better practices and/or attitudes are likely to self-select themselves into the sample, compromising the representativeness of the sample.

## Conclusions

The study concludes that DS use is a prevalent phenomenon in Asir region among middle aged and older adults. Attitudes towards DS is also largely positive. The study highlights the higher DS usage and more positive attitudes among older adults compared to middle aged adults. Multivitamins were the most common type of DS consumed in both age groups. Most participants reported that they use DSs "to maintain good health". The higher DS use is reported among males and married individuals in both age groups and associated with a significant adoption of health-promoting behaviors such as daily physical activity, adequate sleeping patterns, avoiding overweight and obesity and not smoking, all of which are usually cultivated as part of a healthy lifestyle. There is a need to develop an integrated and holistic program to provide health education and disseminate correct scientific DS knowledge regarding the use of dietary supplements and their effects on health. Further studies are needed to assess the factors associated with using prescribed micronutrients and over-the-counter drugs among community-dwelling older adults.

## Supporting information

S1 Checklist STROBE statement—checklist of items that should be included in reports of observational studies. (DOCX)
